# Perspective: Ferromagnetic Liquids

**DOI:** 10.3390/ma13122712

**Published:** 2020-06-15

**Authors:** Robert Streubel, Xubo Liu, Xuefei Wu, Thomas P. Russell

**Affiliations:** 1 Materials Sciences Division, Lawrence Berkeley National Laboratory, Berkeley, CA 94720, USA; wuxuefei06@163.com (X.W.); tprussell@lbl.gov (T.P.R.); 2 Department of Physics and Astronomy, University of Nebraska-Lincoln, Lincoln, NE 68588, USA; 3 Beijing Advanced Innovation Center for Soft Matter Science and Engineering, Beijing University of Chemical Technology, Beijing 100029, China; 4 CAS Key Laboratory of Bio-Inspired Materials and Interfacial Science, Technical Institute of Physics and Chemistry, Chinese Academy of Sciences, Beijing 100190, China; 5 Polymer Science and Engineering Department, University of Massachusetts, Amherst, MA 01003, USA; 6 WPI–Advanced Institute for Materials Research (WPI-AIMR), Tohoku University, 2-1-1 Katahira, Aoba, Sendai 980-8577, Japan

**Keywords:** ferromagnetic liquids, magnetism in curved geometries, self-assembly, magnetic nanoparticles, liquid robotics

## Abstract

Mechanical jamming of nanoparticles at liquid–liquid interfaces has evolved into a versatile approach to structure liquids with solid-state properties. Ferromagnetic liquids obtain their physical and magnetic properties, including a remanent magnetization that distinguishes them from ferrofluids, from the jamming of magnetic nanoparticles assembled at the interface between two distinct liquids to minimize surface tension. This perspective provides an overview of recent progress and discusses future directions, challenges and potential applications of jamming magnetic nanoparticles with regard to 3D nano-magnetism. We address the formation and characterization of curved magnetic geometries, and spin frustration between dipole-coupled nanostructures, and advance our understanding of particle jamming at liquid–liquid interfaces.

## 1. Introduction

Liquid–liquid interfaces play an important role in physical, chemical and biological sciences as they break inversion symmetry and promote the interfacial self-assembly of monolayers of surfactants, colloids and nanoparticles to reduce the interfacial energy [1]. Consequently, the interface can be effectively functionalized by the segregation of molecular surfactants [2,3], polyelectrolytes [4,5], biomaterials [6], liquid crystals [7,8] and micro/nanoparticles [1,9], to the interface endowing the interface with the inherent characteristics and functionalities of these materials. Provided the binding energy of the particles to the interface is sufficiently high, immiscible liquid phases can be emulsified and stabilized against coalescence, affording compartmentalization that enables mass or ion transport for drug delivery, or fluidic reactors [10,11]. Depending on the inherent properties of the particles, the interfaces can be made responsive to magnetic [12,13], optical [14,15,16], electric or mechanical fields, or chemical and biological stimuli [17,18,19].

In general, the binding energy of nanoparticles to an interface is, due to the particle size, insufficient to prevent the ejection from the interface upon mechanical compression, which causes the interfacial area and, therefore, the shape to relax to the equilibrium spherical geometry. A sufficiently large binding energy of the particles, be they soft or hard, can be achieved by increasing particle size or providing a Janus-type of heterogeneous surface functionality. In such cases, the monolayer assemblies can support an in-plane compressive load and are not ejected from the interface. For particles with a uniform size distribution, this compression leads to either a crystallization or vitrification of the 2D assemblies that arrests long-range motions of the particles [20,21,22,23,24]. When nanoparticles in one fluid phase interact with polymeric ligands in a second immiscible fluid phase, the ligands anchor to the surface of the particle exposed to the ligand solution, forming nanoparticle surfactants where the binding energy of the nanoparticles to the interface is significantly enhanced. Consequently, fluid domains with shapes far different from spherical can be locked in by the jamming of the nanoparticles at the interface. During the system’s attempt to reduce the interfacial area the areal density of the nanoparticle surfactants increases and a percolated pathway of nanoparticle surfactants forms that can bear the in-plane compressive load, arresting any further changes to the shape of the domains [25]. Hence, the liquids can be structured. The interfacial assemblies of the nanoparticle surfactants provide means to manipulate both shape and locomotion of the confined droplet.

Recently, it was shown that the jammed interfacial assembly of superparamagnetic nanoparticles, encapsulating a fluid dispersion of superparamagnetic nanoparticles, transforms the original ferrofluid [26] into a ferromagnetic liquid droplet [13]—a fully liquid system with solid-state ferromagnetic properties that can be reconfigured. The magnetic characteristics of these droplets allow for studying structural transitions of the assemblies, i.e., the transformation of the assemblies from a 2D liquid state into a 2D jammed glassy state, and enable us to explore spin glass materials at 2D curved liquid–liquid interfaces. This perspective gives a brief overview of recent progress in generating and characterizing structured liquids and discusses future directions, challenges and application potentials of ferromagnetic liquids.

## 2. Structuring Liquids

Hard colloidal suspensions exhibit a concentration dependent vitrification at which the dispersion reaches a state where the volume density of the dispersed particles is sufficiently high to retard the long-range movement of single particles [20,21,22,23,24]. If the densification process is done rapidly, the dispersion can densely pack into a disordered state, i.e., structural glass, where the relaxation times of the particles is much longer than the observation time. Such vitrified systems have long relaxation times and will densify or age with time. This is the classic behavior of glassy materials. However, if, during the densification, a percolated pathway is formed by the particles that can support a load, the dispersion will jam and further volume relaxations are arrested. Particles that are not jammed can still exhibit short-range motions, but the entire dispersion is locked in volume. Sand piles, tectonic plates, hour glasses, grain silos and granular matter, in general, exhibit such behavior. A similar behavior is seen in 2D assemblies of particles at interfaces where the interfacial jamming of particles can arrest the change in shape of a fluid, since the system attempts to minimize the interfacial area, analogous to volume in 3D systems [25]. Bicontinuous jammed emulsions or “bijels”, a concept introduced by Cates and Clegg, is one example where the assembly of particles at the interface between two fluids undergoing spinodal phase separation can lock in a bicontinuous morphology as the systems attempts to reduce the interfacial area [22].

Generally, nanoparticles functionalized with a uniform coverage of ligands to disperse the nanoparticles in a liquid, will segregate to the interface with a second immiscible liquid to reduce the interfacial energy, regardless of the nanoparticle size and shape. The assemblies are dynamic, i.e., nanoparticles can adsorb and desorb from the interface. For instance, liquids, which are driven away from their spherical equilibrium shape by an external field, will upon removal of the field, eject nanoparticles from the interface until a spherical shape of the liquid phase is attained, since the binding energy holding the nanoparticles to the interface is, due to their size, insufficient to support an aspherical shape. We found that a uniform functionalization of nanoparticles dispersed in one liquid and placed in contact with a second immiscible liquid containing ligands with a complementary functionality, enables the formation of nanoparticle surfactants at the interface by anchoring ligands to the nanoparticles. The number of ligands that attach to the nanoparticles is self-regulated to minimize the interfacial energy per particle. This behavior was discovered by Cui et al. using silica particles, functionalized with carboxylic acid groups in water and brought in contact with a silicon oil solution with polystyrene ligands; the electrostatic interactions between the ligands and the nanoparticles led to the nanoparticle surfactants. When an electric field was placed across the droplet, the dielectric constant difference between water and oil deformed the spherical droplet into an ellipsoid that, upon removal of the electric field retained its shape, owing to the jamming of monolayer nanoparticle surfactants at the interface [18].

**Figure 1:**
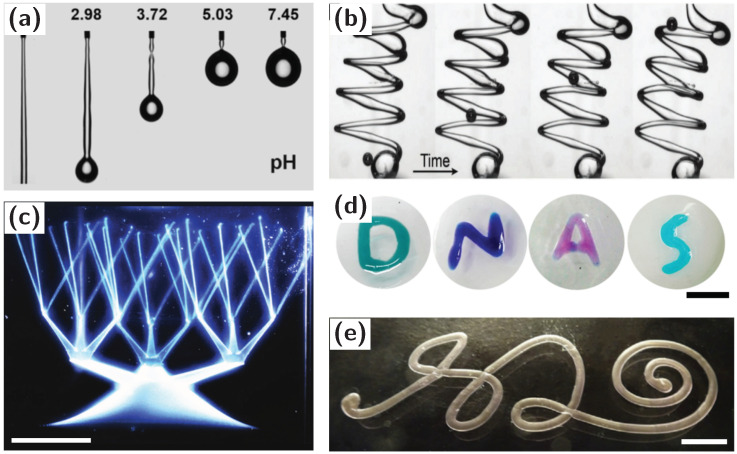
3D printing of structured liquids. (**a**) Break-up length variation of an aqueous cellulose nanocrystals solution falling in toluene that contains PS-NH${}_{2}$ with different pH values. The Plateau–Rayleigh instability effect is greatly inhibited by the stronger electro-static attraction force between the polymer ligand and nanoparticles at lower pH. (**b**) 3D printing of nanoparticle surfactant-stabilized aqueous threads in a silicone oil. (**c**) Complex patterns can be printed into granular gel media by injection using microscale capillary tips. Scale bar is 10 mm. (**d**) DNA surfactants molded by the interfacial jamming of DNAS. Scale bar is 10 nm. (**e**) 3D printing of water in water construct stabilized by an elastic polyanion-polycation coacervate membrane. Scale bar is 5 mm. Figures adapted from References [4,6,27,29,30].

The application of an external electric or shear field to the fluid phases breaks the jamming of the nanoparticle surfactants and enables the reshaping of liquids; more nanoparticle surfactants form and assemble at the interface, thereby locking in a different shape of the liquids. This process can be repeated to arbitrarily structure the liquids. In other words, we have a situation where liquids retain all of their liquids characteristics but can be shaped like a solid. This approach has been generalized to 3D printing of one liquid in another, molding of one liquid in another, fabricating 2D and 3D microfluidic devices and encapsulated reactor systems, having applications in liquid microfluidics, liquid reactors, all-liquid tissue engineering and liquid biomimetic devices. The formation and assembly of nanoparticle surfactants at the interface of an aqueous phase being jetted into an oil reduces the interfacial energy and, hence, suppresses the Plateau–Rayleigh instability enabling 3D printing of one liquid phase in another (Figure 1a,b) [27,28]. The combination of particle jamming and self-healing of existent structures upon penetration with a needle to inject the fluid provides means to synthesize branched structures with T-junctions, interlocked tubules and other complex, interconnected liquid assemblies and devices [29]. Polyelectrolyte surfactants, such as sodium carboxymethyl cellulose surfactant, dissolved in water, have been used to print spiral structures in toluene [5]; granular gels afford a means to devise hierarchical constructs (Figure 1c) [30]. Liquid molding based on highly interfacial active CNC-surfactants were used to produce a range of different geometries of aqueous phases in an oil phase (Figure 1d) [6,31]. Low viscosity liquids enable the rapid fabrication of liquid constructs with complex interconnected structures. Molded channels have been employed for selective mass transportation based on modifying the walls with cationic or anionic molecules, enzymes and colloidal nanocrystal catalysts [11]. A multi-responsive structured liquid system can be used to dynamically control droplet reactors [10]. It should be noted that the interfacial formation and jamming of the nanoparticle surfactants is not constrained to oil/water systems, aqueous/aqueous and oil/oil systems are possible (Figure 1e) [4,32].

**Figure 2:**
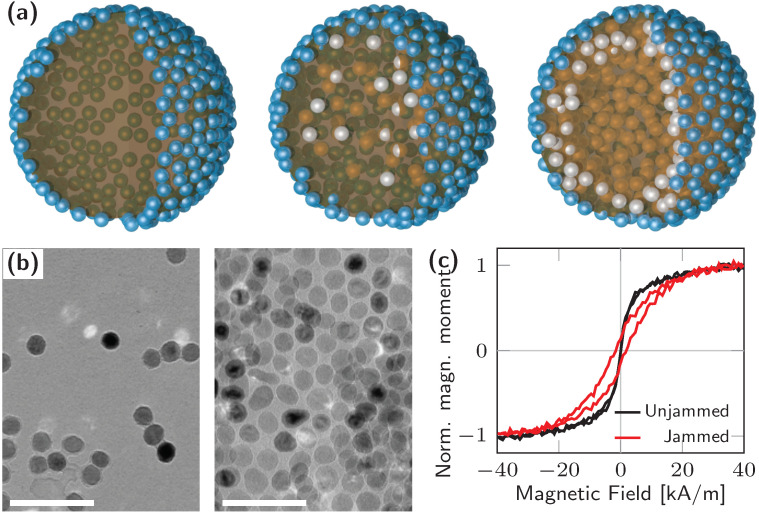
Jamming of superparamagnetic nanoparticles at liquid–liquid interfaces. (**a**) Schematics of ferromagnetic liquid droplets structured by jammed nanoparticles (blue spheres) without and with dispersed nanoparticles (gray spheres), and with nanoparticle surfactants forming multilayers (gray spheres). (**b**) Structural short-range order of unjammed and jammed superparamagnetic nanoparticles visualized with transmission electron microscopy. Scale bar is 100 nm. (**c**) Magnetic hysteresis loops of jammed (ferromagnetic) and unjammed (paramagnetic) liquid droplets obtained by vibrating sample magnetometry.

Consider now a ferrofluid consisting of 20 nm-sized iron oxide (Fe${}_{3}$O${}_{4}$) nanoparticles functionalized with carboxylic acid groups dispersed in water. When a droplet of this ferrofluid is placed in an oil phase, the inherent negative charge at the oil/water interface prevents the negatively charged iron oxide nanoparticles from assembling at the interface. In the presence of a strong magnet, the aqueous phase will assume a shape with pronounced spikes, due to a balance between the magnetic force acting on the particles and the interfacial energy. Dissolving polymeric ligands with a complementary amine-group functionality in the oil phase causes monolayers of ligand surfactants at the oil/water interface. The functionalized nanoparticles will diffuse to the interface, interact with the ligands and form magnetic nanoparticle surfactants. Similar to the non-magnetic particles, the binding energy per particle can be tuned by pH to promote the formation of a shell of jammed magnetic nanoparticles at the interface which encapsulates the aqueous dispersion of the nanoparticles (Figure 2a). Surprisingly, upon application of an external magnetic field, the ferrofluid droplet transforms into a ferromagnetic liquid droplet. The magnetic moment and magnetic anisotropy originate from the dipole coupling among disordered nanoparticle surfactants. If the assembly of the superparamagnetic nanoparticles are unjammed, the droplet reverts to a ferrofluid (Figure 2c).

**Figure 3:**
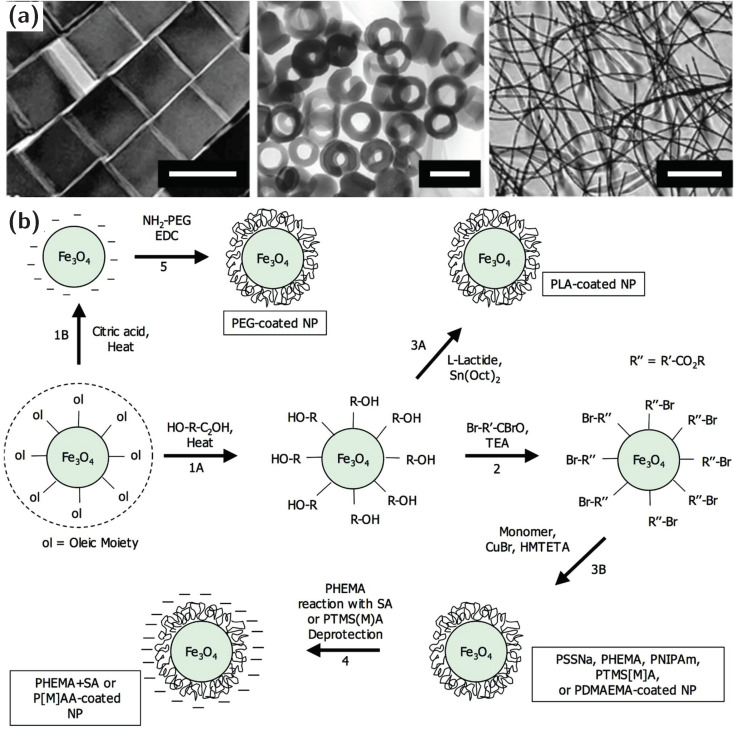
Functionalized magnetic nanoparticles for synthesizing ferromagnetic liquids. (**a**) Selection of iron oxide nanoparticle shapes: nanocubes, nanorings and nanowires, visualized with transmission electron microscopy. Scale bar (from left to right): 100 nm, 200 nm, 30 μm. (**b**) Ligand functionalization of nanoparticles to promote interfacial assembly: 1A and 1B: ligand exchange reactions; 2: acylation of hydroxyl groups to prepare atom transfer radical polymerization (ATRP) surface initiators; 3A: surface-initiated ring opening polymerization of L-lactide; 3B: surface-initiated ATRP; 4: deprotection or additional reaction after polymerization; 5: grafting of end-functionalized polyethylene glycol (PEG) chains onto the nanoparticle surface using amidation chemistry. Figures (**a**,**b**) adapted from References [33,34,35,39].

The wide use of magnetic nanoparticles in biomedical research, in vivo targeted drug delivery, magnetic hyperthermia, radiological contrast agents for imaging and biomolecular separations, to name a few, stimulated the synthesis of magnetic nanoparticles with a large range of sizes and shapes, including cubes [33], rings [34] and wires [35] (Figure 3a). This provides a large variety of materials to tailor their interfacial behavior. Of the three different classes of magnetic nanoparticles, metal oxide (iron oxide), metal (nickel) and metal alloy (iron-platinum, cobalt-platinum), metal oxides and alloys are generally stable in dispersed solutions which is essential to construct ferromagnetic liquids. These particles are typically synthesized by chemical deposition [36] like alkaline solution precipitation, thermal decomposition, microwave heating methods, sono-chemical and electro-physical techniques [37], and spray pyrolysis [38]. The challenge with synthesizing nanoparticles is to provide monodisperse, water-soluble magnetic nanoparticles, such as oleic acid-stabilized magnetic nanocrystals, through e.g., seeded mediated growth. The stabilizing peripheral ligands on the nanoparticle surface can be replaced by ligand exchange reactions with capping agents bearing reactive hydroxyl moieties (Figure 3b) [39]. The hydroxyl groups initiate ring opening polymerization (ROP) of polylactic acid and esterify by acylation to permit the addition of alkyl halide moieties and initiate the atom transfer radical polymerization (ATRP). Finally, the monodisperse magnetic nanoparticles are coated by a stabilizing polymer, such as polylactide (PLA), polyethylene glycol (PEG), poly(styrenesulfonate) (PSS), polyacrylic acid (PAA) and poly(N-isopropylacrylamide) (PNIPAM).

## 3. Magnetism of Ferromagnetic Liquids

The magnetic properties of ferromagnetic liquids are governed by the jammed interface comprised of millions of nanoparticles with slightly different size and shape. Each nanoparticle is uniformly magnetized and resembles a macro spin. The net magnetic moment of isolated specimens vanishes at remanence owing to thermal spin fluctuations in the form of a random rotation or flipping of the macro spin. This is because shape and magneto-crystalline anisotropy are insufficient to establish a large energy barrier necessary for stable room-temperature configurations. Dispersed nanoparticles possess additional translational and rotational degrees of freedom triggered by Brownian motion (thermal excitation), magnetic field (torque) or field gradients (initial torque and translation). Individual superparamagnetic nanoparticles become ferromagnetic due to magnetic stray field interactions when separated by a few nanometers, similar to planar nanodisks [40]. This condition is only satisfied by jamming nanoparticles at the liquid–liquid interface since aggregation in the dispersion is hindered by charged ligands (Figure 2b,c) [13]. The magnetization configuration, coercive fields and magnetic stability of the nanoparticle surfactants depend on the assembly’s short-range order and magnetic dipole coupling, and are, for μL and nL volumes due to magnetic short-range ordering, independent of droplet shape and size. Additionally, the spatial confinement of thermally stable macro spins to the liquid–liquid interface results in a small in-surface anisotropy and a resemblance of dipolar spin systems with XY or spin glass character. In contrast to conventional dipolar spin systems, such as 2D planar artificial spin ice structures [41], the present systems are defined on 2D curved and closed surfaces that can be controlled by chemical means.

**Figure 4:**
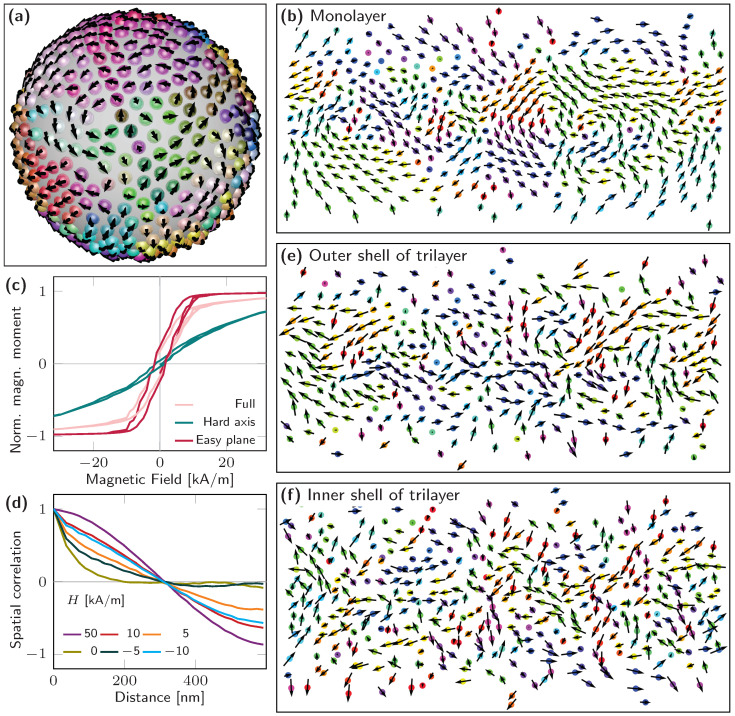
Magnetization configuration of XY macro-spins jammed on a curved 2D spherical shell. (**a**) 3D view of remanent state of monolayer nanoparticle surfactants (Fe${}_{3}$O${}_{4}$: Ø=25 nm, $M_{s}=300$ kA/m, T=0 K) stochastically positioned on sphere with mean distance of 30 nm (Fe${}_{3}$O${}_{4}$ plus ligand shell). Black arrows indicate direction and magnitude of tangential magnetization components. Color refers to polar angle of magnetization vector. (**b**) 2D view (ϕ vs. θ) of remanent magnetization projected onto curved surface revealing magnetization reversal via vortex nucleation. Color highlights in-plane magnetization direction. (**c**) Magnetic hysteresis loops for entire magnetic system, and easy plane and hard axis regions. The in-surface anisotropy originates from magnetic dipole coupling between adjacent nanoparticles that manifests a remanent magnetic moment. (**d**) Spatial spin-spin correlation calculated for 2D system, i.e., not in 3D space, at various magnetic fields revealing magnetic short-range order at remanence (vortices and domains) and long-range order in the presence of external magnetic fields. (**e**,**f**) 2D views (ϕ vs. θ) of remanent magnetization for trilayer nanoparticle surfactants demonstrating substantially weaker dipole coupling perpendicular to the interface and reduced in-surface anisotropy and magnetic order.

This qualitative understanding, reported in Reference [13], gives a first insight into the magnetism of ferromagnetic liquids based on jamming nanoparticles. However, the close relation between structural order and magnetic properties calls for a quantitative exploration of the physical mechanisms to address small nuances, improve synthesis capabilities and realize aspiring liquid magnetic robotics. Even ideal spherical nanoparticles (isotropic macro spins), rather than the more realistic aspherical nanoparticles, show a high affinity toward structural disorder of coercive field, saturation field, magnetic moment and magnetization configuration. These aspects can be examined by modeling dipole-coupled macro-spin systems confined to a spherical shell (Figure 4). The combination of structural short-range order and geometric frustration manifests a magnetic short-range order that becomes obvious, particularly near remanence, in the form of vortex nucleation along the equator and at the poles when applying an external magnetic field (Figure 4b). The normalized remanent magnetization and magnetic susceptibility $\frac{dM}{dH}$ are independent of the magnetic moment of individual nanoparticles, and depend on the short-range order and layer thickness of the jammed interface. The latter two define the in-surface magnetic anisotropy, reflected by easy plane and hard axis hysteresis loops (Figure 4c), that, to a great extent, prevents at remanence magnetic dipole coupling between different layers in multilayer shells (Figure 4e,f). In the presence of a magnetic field, multilayer nanoparticle surfactants are magnetically softer than monolayers.

Although nanoparticles and self-assemblies have been extensively studied for various reasons in the past, investigations of ferromagnetic liquids are challenging, as conclusions drawn from dried structures do not necessarily reflect the structural and magnetic properties of the liquid system. This is true for electron, x-ray and scanning probe microscopies to assess structural short-range ordering, and magnetometry and magnetic imaging to probe the magnetization configuration. Vibrating sample magnetometry was used to measure the magnetic hysteresis loops of the entire systems, and categorize ferromagnetic liquids (jamming) and ferrofluids (unjamming) (Figure 2c). By these means, it was shown that the magnetic properties of μL droplets do not depend on droplet shape and size [13]. This behavior is consistent with short-range correlations of macro spins at the jammed interface and a magnetic dipole-mediated synchronization between jammed and dispersed nanoparticles. A differentiation between easy plane and hard axis magnetization reversal to quantify coercive and saturation field can be given by locally probing the magnetic hysteresis loop with magneto-optical Kerr effect magnetometry.

**Figure 5:**
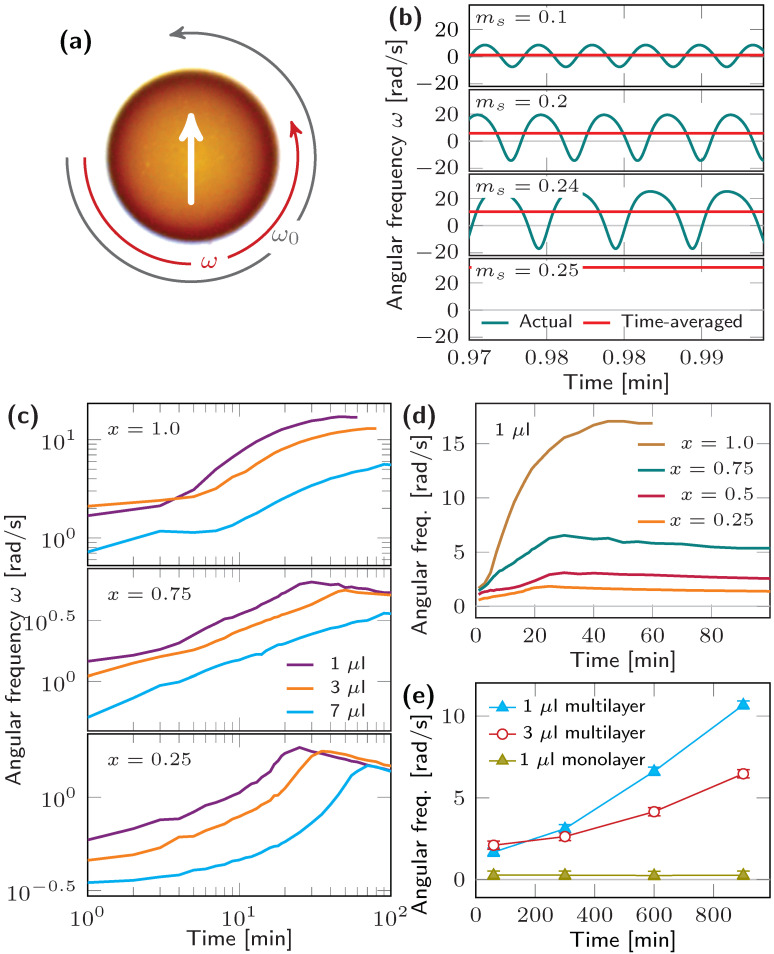
Magnetic field-driven gyration of ferromagnetic liquid droplets. (**a**) Droplet rotates with angular frequency ω to align magnetic moment along the external magnetic field, driven at $\omega_{0}=2\pi \times 5$ Hz. (**b**) Modeling of 1 μL droplet revealing friction-dominated desynchronized gyration for insufficient torque, and a reduction of the time-averaged experimental value due to periodically altering acceleration. (**c**) Experimental data for multilayer nanoparticle surfactants consisting of various (Fe${}_{3}$O${}_{4}$)${}_{x}$:(SiO${}_{2}$)${}_{1-x}$ nanoparticle mixtures showing two different regimes where angular acceleration increases and decreases. (**d**) Angular frequency for 1 μL droplets with different *x*. (**e**) Growing multilayer and monolayer nanoparticle surfactants reveal monotonically increasing and constant angular frequency, respectively, both in experiment and modeling. Time t=0 indicates droplet formation.

A different perspective can be obtained from magnetic field-driven hydrodynamics experiments involving translation and rotation of ferromagnetic μL liquid droplets. This approach is simple yet powerful and takes advantage of a frozen/locked magnetic moment to study the three different phases related to the formation of ferromagnetic liquids: migration of nanoparticles to the interface, jamming and equilibrium hydrodynamics of jammed interfaces. Tracking the angular frequency as a function of time and driving frequency of a rotating magnetic field, droplet shape or viscosity allows for retrieving information about the magnetic moment of the droplet (Figure 5). Aside from an initial onset due to friction, a constant magnetic moment, i.e., jammed interface thickness, leads to a constant time-averaged angular frequency, which equals the driving frequency upon synchronized gyration (Figure 5b). An insufficient torque (magnetic moment) causes a desynchronized rotation with regular clockwise and counterclockwise acceleration that significantly reduce the experimentally accessible, time-averaged angular frequency. Lowering friction, i.e., reducing droplet size or viscosity, or driving frequency can compensate for a low magnetic moment. In other words, a small driving frequency can lead to a faster rotation of the ferromagnetic liquid droplet. The nanoparticles migration to the interface results in a prolonged onset that is particularly prominent in solutions with high pH (enhanced electro-static shielding) or low particle concentration. A monotonically increasing angular frequency implies a growing magnetic moment that originates from a jammed surfactant layer beyond the commonly understood monolayer structure, i.e., bilayer and multilayer (Figure 5c–e). Partial substitution of magnetic nanoparticles with non-magnetic specimens does not only lower the angular frequency, owing to a smaller magnetic moment, but also affects the temporal evolution which manifests in an increased or decreased time-averaged angular acceleration (Figure 5c). The increased acceleration for low iron oxide particle concentrations is attributed to a preference of clustering of magnetic and non-magnetic nanoparticles during surfactant rearrangement, replacement and adsorption. This selection is driven by dipole attraction and enhances the magnetic moment. To date, it is unclear whether the formation of bilayers and multilayers is unique to ferromagnetic liquids due to magneto-static attraction or universal to jammed interfaces. Although mechanical properties, such as wrinkling and rigidity, of jammed interfaces of non-magnetic nanoparticles coincide with those expected for multilayers, experimental evidence can only be given by visualizing the spatial distribution of nanoparticles near the liquid–liquid interface with tomographic imaging harnessing x-rays or electrons, or neutron reflectometry.

## 4. Scientific Perspective

**Figure 6:**
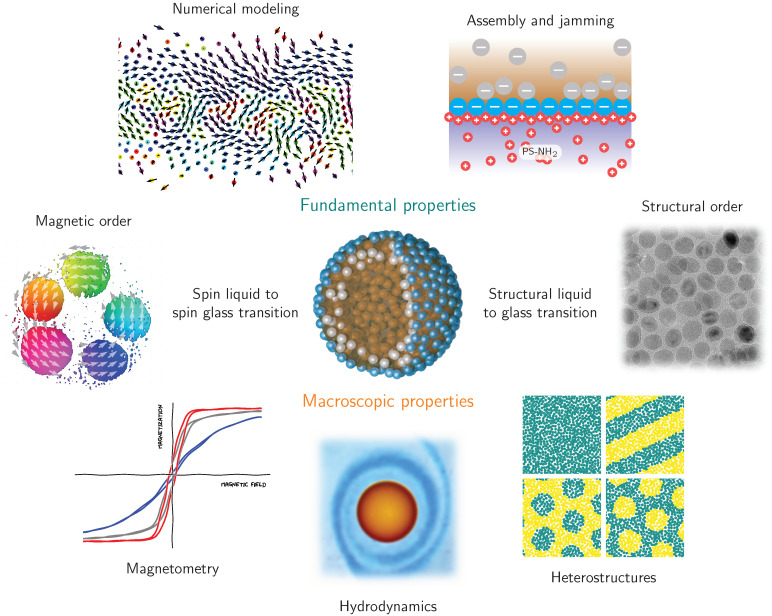
Scientific perspective on ferromagnetic liquids concerning the correlation between structural and magnetic short-range order, and the link between fundamental and macroscopic properties. Advanced x-ray, electron and neutron techniques to probe assembly and jamming of nanoparticles on characteristic length and time scales will corroborate predictions retrieved from molecular dynamics, Monte Carlo and micromagnetic simulations. Local magnetometry and hydrodynamics studies will aid in assessing macroscopic properties.

The magnetic functionalization of structured liquids is a milestone toward designing liquids with properties previously presumed to be unique to solid-state materials. In order to mature ferromagnetic liquids and realize their application potential for liquid robotics, targeted drug delivery, liquid optical components, functional microfluidic channels and liquid magnetic sensing, it is essential to adapt current state-of-the-art characterization techniques and synthesis approaches. We envision that scientific efforts will focus on the jamming process of nanoparticles at liquid–liquid interfaces, spin frustration and thermal spin fluctuations in 2D curved dipolar spin glass materials, and magnetization configurations in structured liquids with distinct shapes and various nanoparticles properties (Figure 6).

To date, the characterization of magnetic properties has heavily been based on vibrating sample and magneto-optical Kerr effect magnetometry revealing integral and local information about the magnetic hysteresis loops. This approach can be used to distinguish jamming from unjamming states and to survey a large parameter space. The compatibility of Kerr magnetometry with micro- and nano-fluidics will enable swift studies of the influence of chemical, geometric and structural parameters on magnetic properties without changing the experimental setup. Magnetic field-driven hydrodynamics experiments of gyrating μL droplets will complement these investigations by offering insight into the temporal evolution of jamming and the thickness of the magnetic interface. Current analytical approximations of the hydrodynamics need to be advanced to improve agreement with experimental data. Switching from micromagnetic to Monte Carlo simulations to properly treat finite temperature effects, such as thermal fluctuations, will resolve the discrepancy between numerical and experimental values for coercive and saturation fields, while simultaneously speeding up computations. The theoretical treatment of geometric spin frustration in spin glasses on 2D curved surfaces with different shapes and topologies will guide future experiments harnessing advanced x-ray and electron tools. This pertains in particular to a systematic study of spin liquid to spin glass phase transitions mediated by structural liquid to glass transformation as a function of temperature, short-range order, particle shape, magneto-crystalline anisotropy and magnetic moment, as well as of the magnetization reversal via homogeneous states, random spins or vortex and string nucleation.

The common theme of the aforementioned approaches is the inference of structural ordering of nanoparticles from magnetic properties. A significant step will be to probe structural and magnetic order on the tens of nanometer scale. This includes in particular spatial and temporal correlations of nanoparticles at liquid–liquid interfaces and corresponding macro spins on different time scales harnessing resonant coherent x-ray scattering [42] and small-angle neutron scattering and reflectometry [43], and visualization of stable jammed interfaces with microscopy and tomography [44,45,46,47,48]. The short attenuation length of soft x-rays and electrons limits experiments to thin bilayers of two liquids with similar density and few layers of nanoparticles, sandwiched between silicon nitride nano-membranes or graphene sheets. While electron holography with transmission electron microscopy stands out by a superior spatial resolution (Δx∼1 nm), x-rays (Δx∼20 nm) shine through an element specific contrast based on the x-ray magnetic circular dichroism effect. Additionally, electrons probe the in-plane components of the magnetic induction and x-rays the normal magnetization component. Thicker films and even entire liquid droplets can be examined with hard x-rays and polarized neutrons. The larger penetration depth is related to a weaker attenuation/absorption, which requires a substantially longer data acquisition. In contrast to direct imaging with conventional microscopy, scattering-based characterization probes spatial and temporal correlations, and allows for studying systems whose structural and magnetic properties vary with time. The latter is key to small-angle neutron scattering and reflectometry that typically averages over an area of 1 cm${}^{2}$ and provides information about the depth profile of structural short-range order and, in combination with spin-polarized neutrons, the magnetization configuration. Since the structural short-range order and density of nanoparticle surfactant layers are expected to vary only slightly within the interface, neutron scattering and reflectometry have great potential to reconstruct the depth profile with nanometer resolution from the averaged data recorded at varies incidence angles.

The jamming process requires a sufficient number of nanoparticles; before that, nanoparticles adsorb to, move along and detach from the liquid–liquid interface. Theoretically, insight into the assembly and jamming of nanoparticles at liquid–liquid interfaces can be obtained from molecular dynamics simulations. Knowledge about the short-range order of jammed nanoparticles is particularly crucial to micromagnetic and Monte Carlo simulations, which currently rely on stochastically distributed nanoparticle locations. Molecular dynamics will further reveal the impact of internal and external magnetic fields on the self-assembly of superparamagnetic nanoparticles, including means to tailor structural short-range order and magnetic properties of ferromagnetic liquids. Another critical aspect is the interplay between magnetic and non-magnetic nanoparticles during the assembly and jamming process, causing a possible clustering, which is essential to analytical studies and comparison with experiment. The time scales depend both on the age of the droplet and chemical/structural parameters. The current experimental capabilities of synchrotron facilities (minutes to seconds) will be pushed toward milli- and microseconds by diffraction-limited coherent synchrotrons, which are currently under development and scheduled to launch toward the end of this decade. Nano- to picoseconds time scales are accessible with free electron lasers. Access to the micro- and nanosecond time scale will allow for exploring early stages of adsorption and motion of nanoparticles at the interface, the evolution of structural and magnetic order and how they relate to each other, as well as spin frustration and fluctuations in weakly coupled macro spin systems. Understanding the mechanism and time scales of self-healing of droplets is essential to mechanical or field-induced deformation that appears to preserve the magnetic moment.

Similarly interesting is the question of self-assembly in the presence of a magnetic field, such as external fields and stray fields emanating from adjacent nanoparticles. Modifying the magnetic dipole coupling can be achieved by changing temperature, magnetic core diameter and non-magnetic shell thickness, or mixing non-magnetic and superparamagnetic particles. Aside from a preferential chain formation of adjacent nanoparticles along the direction of the external magnetic field, assembly and jamming of mixed phases in large magnetic fields will enable patterning and heterostructuring of nanoparticle surfactant layers whose surface normal is parallel to the field direction (Figure 6). The formation of disordered labyrinths, 1D stripe domains and trigonal lattices is prominent for superparamagnetic nanoparticles with diameters of ≲10 nm that form only a ferromagnetic interface in the presence of an external magnetic field, similar to ferrofluids and solid-state thin films with perpendicular magnetic anisotropy [49]. A magnetic stray field favoring spatially dependent idle times of nanoparticles at the interfaces prior to jamming could allow for tailoring symmetry and periodicity of the magnetic nanoparticle surfactant layer. These nanopatterned interfaces possess distinct optical properties owing to different refractive indices that may serve as configurable birefringent, diffractive and even chiral materials.

The fundamental understanding of ferromagnetic liquids will facilitate printing of a large variety of complex, structured liquids with engineered structural and magnetic properties. This includes varying ligand functionalization and length to stabilize monolayers, bilayers, trilayers and multilayers of jammed interfaces at different liquid–liquid interfaces, including water/oil, oil/oil, water/water and conductive liquid/non-conductive liquid. Low-temperature experiments to vary thermal spin fluctuations and Brownian motion may be carried out at oil/oil interfaces without causing physical damage due to freezing. The initial emphasis on spherical and aspherical droplets, tubes and jets to investigate fundamental properties will eventually shift toward more complex geometries, including connected networks, concentric cylinders, linked tori and Möbius bands. These systems can be used to study inversion symmetry breaking, spin chirality selection and curvature effects [50,51,52] in 2D curved dipole-coupled systems from the perspective of artificial spin ice structures [41], topological states and spin wave propagation. Dipole-coupled systems have successfully been applied to resemble spin frustration of exchange-coupled materials [41]; theoretically predicted magneto-chirality effects [53] emerge from curvature and inversion symmetry breaking in exchange-coupled systems, and stabilize skyrmionic spin textures in e.g., spherical shells [54,55]. It is highly interesting to determine the extent to which ferromagnetic liquids as a representative of curved dipole-coupled systems can resemble exchange-coupled magnetic materials in view of both static and dynamic properties.

## 5. Technological Perspective

**Figure 7:**
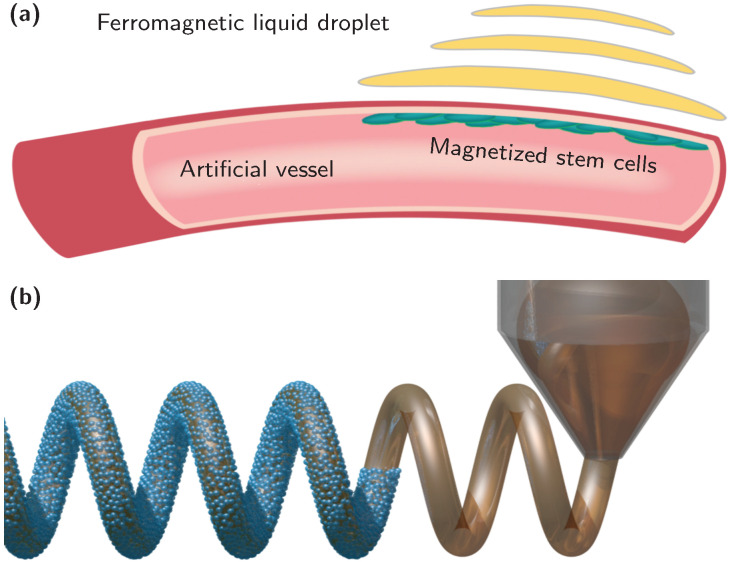
Technological perspective of ferromagnetic liquid droplets. (**a**) Adaptive directional tissue growth stimulated by magnetic fields of ferromagnetic liquids. (**b**) 3D printed ferromagnetic liquid components with arbitrary shape for magnetic-remote-controlled robotic devices.

Technology-oriented applications of ferromagnetic liquid droplets take advantage of a controlled generation of local magnetic fields to promote adaptive directional tissue growth, and the structural and magnetic reconfigurability to design configurable optical components, functional microfluidic channels for particle, drug and cell transport, sensors and actuators (Figure 7). Magnetically powered devices, such as electromagnetic coils, magnetic fluid seals and permanent magnet equipment, are typically fabricated from rigid, hard-condensed matter. The result is a magnetic field with a specific strength and spatial distribution. In this sense, ferromagnetic liquids open up an entirely new world of magnetic materials benefiting from mechanical and magnetic reconfigurability; for instance, 3D printed water-in-oil magnetic liquid tubules offer arbitrary shape (fluidity) and self-adaptive permanent magnetization (solidity), which benefit magnetic liquid actuators, non-contact liquid capsule delivery and other magnetically controlled devices in all-liquid environment.

Magnetically driven autonomous technology often relates to the parallelized and sequential operations with respect to actuators in medical diagnostics, non-contact delivery and chemicals synthesis [56,57,58,59,60,61,62,63]. Reconfigurable ferromagnetic liquids representing magnetic liquid actuators and sensors, interact with the external magnetic field to exert translation and precession movement that can be facilitated in analogy to solid-state magnets. The magnetic field-sensitive patterning of superparamagnetic nanoparticles and of mixed phases of non-magnetic and superparamagnetic nanoparticles offers a mean to design adaptive optical components, such as refractive mirrors, diffractive, birefringent and chiral materials. The remanent magnetization of ferromagnetic liquid droplets may inspire all-liquid sensors, complementing functionality and application of conventional solid-state sensors based on e.g., Hall effect, giant magneto-resistance/impedance and giant stress-resistance/impedance. Tailoring ligand functionalization of aspherical nanoparticles and in-field printing of ferromagnetic liquids will enable manufacturing of tubules with azimuthal, longitudinal or radial magnetization with variable domain size and magnetic susceptibility/coercivity. The latter can be tailored by particles, such as Fe${}_{3}$O${}_{4}$, FePt, Co and Nd-based alloys, with different saturation magnetization, magneto-crystalline anisotropy and heterogeneous ligand functionalization to provide a homogeneous, periodically or locally varying distribution of particles, particle orientation and magnetic fields. Local variations are particularly interesting for actuators and sensors with complex geometries and multiple ranges of operation. While nanoparticles with increased saturation magnetization extend the range of operation toward larger fields due to increased magnetic anisotropy, they may also suffer from dipole attracting and aggregation in their dispersed state. Synthesis at elevated temperatures and larger electro-static repulsion between specimens could prevent this segregation. The overarching questions regarding magnetic sensing with ferromagnetic liquids is the detection of physical changes that is conveniently done by magneto-resistance measurements in exchange-coupled metallic materials. For these applications, electro-chemical plating using a porous alumina template and strain-engineering rolled-up nanotech facilitating intrinsic strain gradients have been employed [60,61] and refined to synthesize nano-rods, nano-tubes and multi-segmented specimens [64,65,66,67,68,69,70] with variable diameter (<50 nm), and microtubules with tailored magnetization configuration [71,72] that achieve unprecedented sensitivity based on the giant magneto-impedance effect [73].

Remote tissue engineering harnessing optical, acoustic and magnetic fields has attracted much attention in the last decades. Examples range from initiating cell differentiation or material degradation, to assembling components for tissue organization, aligning cell and matrix fibers [74]. The directed regeneration of aligned collagen fibers [75] and oriented neural cells requires only small magnetic fields [76], which can be provided by ferromagnetic liquid constructs. The latter could be remotely positioned around the growing cells or organ tissues, either in the form of flowable magnetic emulsion droplets or individual integrated liquid tubule (Figure 7a). Taking advantage of 3D bio-printing, matrices, such as hydrogel containing active stem cells, can be shaped along the ferromagnetic liquid scaffolds ensuring a magnetically aligned cell differentiation and proliferation. This is particularly appealing for blood vessels, cartilage and nerve tissue regeneration. The flexible, yet fragile ferromagnetic liquid structure can be supported by microfluidic devices with dual channels, branched or more complex tubular structures. The integration of both magnetic liquid scaffolds and cell culture matrix enables an adaptive growth of the magnetized tissue with high fidelity.

## 6. Conclusions

Assembly and jamming of nanoparticles at liquid–liquid interfaces is a versatile platform to structure liquids with virtually arbitrary shape and solid-state properties. Their geometries can be reversibly configured by means of mechanical agitation, pH change of the dispersion or electric and magnetic fields. The latter requires a magnetic moment, which is provided by superparamagnetic nanoparticles transforming the original paramagnetic ferrofluid into a ferromagnetic liquid droplet. The structural transition from an interfacial 2D liquid state into a 2D jammed glassy state imposes similar magnetic constraints, i.e., dipole-coupled 2D spin glass materials with short-range order and a remanent magnetic moment. Given the close relation between structural and magnetic properties, magnetic characteristics of these droplets can be employed to explore assembly and jamming of nanoparticles at liquid–liquid interfaces, and study spin glass materials at 2D curved interfaces. In particular, we discussed magnetic characteristics of monolayer versus multilayer nanoparticle surfactants, and mixtures of magnetic and non-magnetic nanoparticles by means of numerical modeling and experiments harnessing magnetometry and hydrodynamics of field-driven droplet motion. These studies were followed by elaborating on the future role of numerical modeling and experimental characterization with x-ray, electron and neutron techniques at large-scale user facilities, as well as tabletop instruments. Based on our current understanding of ferromagnetic liquids, we identified future applications of all-liquid robotics for targeted drug delivery, remote tissue engineering and adaptive optics, taking advantage of controlled generation of local magnetic fields, remote control of translational and rotational motion and field-dependent nanopatterning, respectively. These technological applications will heavily rely on interdisciplinary research linking chemistry, physics, magnetism and bio-engineering.

## References

[1] Pieranski P. (1980). Two-Dimensional Interfacial Colloidal Crystals. Phys. Rev. Lett..

[2] Forth J., French D.J., Gromov A.V., King S., Titmuss S., Lord K.M., Ridout M.J., Wilde P.J., Clegg P.S. (2015). Temperature- and pH-Dependent Shattering: Insoluble Fatty Ammonium Phosphate Films at Water-Oil Interfaces. Langmuir.

[3] Pezron E., Claesson P.M., Berg J.M., Vollhardt D. (1990). Stability of Arachidic Acid Monolayers on Aqueous Salt Solutions. J. Colloid Interface Sci..

[4] Xie G., Forth J., Chai Y., Ashby P.D., Helms B.A., Russell T.P. (2019). Compartmentalized, All-Aqueous Flow-Through-Coordinated Reaction Systems. Chem.

[5] Xu R., Liu T., Sun H., Wang B., Shi S., Russell T.P. (2020). Interfacial Assembly and Jamming of Polyelectrolyte Surfactants: A Simple Route To Print Liquids in Low-Viscosity Solution. ACS Appl. Mater. Interfaces.

[6] Qian B., Shi S., Wang H., Russell T.P. (2020). Reconfigurable Liquids Stabilized by DNA Surfactants. ACS Appl. Mater. Interfaces.

[7] Tran L., Kim H.N., Li N., Yang S., Stebe K.J., Kamien R.D., Haase M.F. (2018). Shaping nanoparticle fingerprints at the interface of cholesteric droplets. Sci. Adv..

[8] Moreno-Razo J.A., Sambriski E.J., Abbott N.L., Hernandez-Ortiz J.P., de Pablo J.J. (2012). Liquid-crystal-mediated self-assembly at nanodroplet interfaces. Nature.

[9] Shi S., Qian B., Wu X., Sun H., Wang H., Zhang H.B., Yu Z.Z., Russell T.P. (2019). Self-Assembly of MXene-Surfactants at Liquid-Liquid Interfaces: From Structured Liquids to 3D Aerogels. Angew. Chem. Int. Ed..

[10] Yang J., Kim J., Kim B., Cho Y.J., Lee J.H., Kim S.K. (2018). Vortex-chirality-dependent standing spin-wave modes in soft magnetic nanotubes. J. Appl. Phys..

[11] Feng W., Chai Y., Forth J., Ashby P.D., Russell T.P., Helms B.A. (2019). Harnessing liquid-in-liquid printing and micropatterned substrates to fabricate 3-dimensional all-liquid fluidic devices. Nat. Commun..

[12] Zhang Z., Jiang Y., Huang C., Chai Y., Goldfine E., Liu F., Feng W., Forth J., Williams T.E., Ashby P.D. (2008). Guiding kinetic trajectories between jammed and unjammed states in 2D colloidal nanocrystal-polymer assemblies with zwitterionic ligands. Sci. Adv..

[13] Liu X., Kent N., Ceballos A., Streubel R., Jiang Y., Chai Y., Kim P.Y., Forth J., Hellman F., Shi S. (2019). Reconfigurable ferromagnetic liquid droplets. Science.

[14] Hojeij M., Younan N., Ribeaucourt L., Girault H.H. (2010). Surface plasmon resonance of gold nanoparticles assemblies at liquid | liquid interfaces. Nanoscale.

[15] Edel J.B., Kornyshev A.A., Urbakh M. (2013). Self-Assembly of Nanoparticle Arrays for Use as Mirrors, Sensors, and Antennas. ACS Nano.

[16] Gu P.Y., Chai Y., Hou H., Xie G., Jiang Y., Xu Q.F., Liu F., Ashby P.D., Lu J.M., Russell T.P. (2019). Stabilizing Liquids Using Interfacial Supramolecular Polymerization. Angew. Chem. Int. Ed..

[17] Whitesides G.M., Boncheva M. (2002). Beyond molecules: Self-assembly of mesoscopic and macroscopic components. Proc. Natl. Acad. Sci. USA.

[18] Cui M., Emrick T., Russell T.P. (2013). Stabilizing Liquid Drops in Nonequilibrium Shapes by the Interfacial Jamming of Nanoparticles. Science.

[19] Fang P.P., Chen S., Deng H., Scanlon M.l.D., Gumy F.R., Lee H.J., Momotenko D., Amstutz V., Cortés-Salazar F., Pereira C.M. (2013). Conductive Gold Nanoparticle Mirrors at Liquid/Liquid Interfaces. ACS Nano.

[20] van Megen W., Pusey P.N. (1991). Dynamic light-scattering study of the glass transition in a colloidal suspension. Phys. Rev. A.

[21] Mason T.G., Weitz D.A. (1995). Linear viscoelasticity of colloidal hard sphere suspensions near the glass transition. Phys. Rev. Lett..

[22] Herzig E.M., White K.A., Schofield A.B., Poon W.C., Clegg P.S. (2007). Bicontinuous emulsions stabilized solely by colloidal particles. Nat. Mater..

[23] Cho E.C., Kim J.W., Fernández-Nieves A., Weitz D.A. (2008). Highly Responsive Hydrogel Scaffolds Formed by Three-Dimensional Organization of Microgel Nanoparticles. Nano Lett..

[24] Mattsson J., Wyss H.M., Fernandez-Nieves A., Miyazaki K., Hu Z., Reichman D.R., Weitz D.A. (2009). Soft colloids make strong glasses. Nature.

[25] Ramsden W., Gotch F. (1904). Separation of solids in the surface-layers of solutions and suspensions (observations on surface-membranes, bubbles, emulsions, and mechanical coagulation). Preliminary account. Proc. R. Soc. Lond..

[26] Zhang X., Sun L., Yu Y., Zhao Y. (2019). Flexible Ferrofluids: Design and Applications. Adv. Mater..

[27] Liu X., Shi S., Li Y., Forth J., Wang D., Russell T.P. (2017). Liquid Tubule Formation and Stabilization Using Cellulose Nanocrystal Surfactants. Angew. Chem. Int. Ed..

[28] Toor A., Helms B.A., Russell T.P. (2017). Effect of Nanoparticle Surfactants on the Breakup of Free-Falling Water Jets during Continuous Processing of Reconfigurable Structured Liquid Droplets. Nano Lett..

[29] Forth J., Liu X., Hasnain J., Toor A., Miszta K., Shi S., Geissler P.L., Emrick T., Helms B.A., Russell T.P. (2018). Reconfigurable Printed Liquids. Adv. Mater..

[30] Bhattacharjee T., Zehnder S.M., Rowe K.G., Jain S., Nixon R.M., Sawyer W.G., Angelini T.E. (2015). Writing in the granular gel medium. Sci. Adv..

[31] Shi S., Liu X., Li Y., Wu X., Wang D., Forth J., Russell T.P. (2018). Liquid Letters. Adv. Mater..

[32] Luo G., Yu Y., Yuan Y., Chen X., Liu Z., Kong T. (2019). Freeform, Reconfigurable Embedded Printing of All-Aqueous 3D Architectures. Adv. Mater..

[33] Kim D., Lee N., Park M., Kim B.H., An K., Hyeon T. (2009). Synthesis of Uniform Ferrimagnetic Magnetite Nanocubes. J. Am. Chem. Soc..

[34] Jia C.J., Sun L.D., Luo F., Han X.D., Heyderman L.J., Yan Z.G., Yan C.H., Zheng K., Zhang Z., Takano M. (2008). Large-Scale Synthesis of Single-Crystalline Iron Oxide Magnetic Nanorings. J. Am. Chem. Soc..

[35] Goubault C., Leal-Calderon F., Viovy J.L., Bibette J. (2005). Self-Assembled Magnetic Nanowires Made Irreversible by Polymer Bridging. Langmuir.

[36] Ansari S.A.M.K., Ficiarà E., Ruffinatti F.A., Stura I., Argenziano M., Abollino O., Cavalli R., Guiot C., D’Agata F. (2019). Magnetic Iron Oxide Nanoparticles: Synthesis, Characterization and Functionalization for Biomedical Applications in the Central Nervous System. Materials.

[37] Kurlyandskaya G.V., Bhagat S.M., Safronov A.P., Beketov I.V., Larrañaga A. (2011). Spherical magnetic nanoparticles fabricated by electric explosion of wire. AIP Adv..

[38] Taylor R.M., Monson T.C., Gullapalli R.R. (2014). Influence of carbon chain length on the synthesis and yield of fatty amine-coated iron-platinum nanoparticles. Nanoscale Res. Lett..

[39] Lattuada M., Hatton T.A. (2007). Functionalization of Monodisperse Magnetic Nanoparticles. Langmuir.

[40] Streubel R., Kent N., Dhuey S., Scholl A., Kevan S., Fischer P. (2018). Spatial and Temporal Correlations of XY Macro Spins. Nano Lett..

[41] Skjærvø S.H., Marrows C.H., Stamps R.L., Heyderman L.J. (2020). Advances in artificial spin ice. Nat. Rev. Phys..

[42] Hannon J.P., Trammell G.T., Blume M., Gibbs D. (1988). X-Ray Resonance Exchange Scattering. Phys. Rev. Lett..

[43] Mühlbauer S., Honecker D., Périgo E.A., Bergner F., Disch S., Heinemann A., Erokhin S., Berkov D., Leighton C., Eskildsen M.R. (2019). Magnetic small-angle neutron scattering. Rev. Mod. Phys..

[44] Pfeiffer F. (2018). X-ray ptychography. Nat. Photon..

[45] Shi X., Burdet N., Chen B., Xiong G., Streubel R., Harder R., Robinson I.K. (2019). X-ray ptychography on low-dimensional hard-condensed matter materials. Appl. Phys. Rev..

[46] Donnelly C., Scagnoli V. (2020). Imaging three-dimensional magnetic systems with x-rays. J. Phys. Condens. Matter.

[47] Midgley P.A., Dunin-Borkowski R.E. (2009). Electron tomography and holography in materials science. Nat. Mater..

[48] Wolf D., Lubk A., Röder F., Lichte H. (2013). Electron holographic tomography. Curr. Opin. Solid State Mater. Sci..

[49] Seul M., Andelman D. (1995). Domain Shapes and Patterns: The Phenomenology of Modulated Phases. Science.

[50] Streubel R., Fischer P., Kronast F., Kravchuk V.P., Sheka D.D., Gaididei Y., Schmidt O.G., Makarov D. (2016). Magnetism in curved geometries. J. Phys. D Appl. Phys..

[51] Fernández-Pacheco A., Streubel R., Fruchart O., Hertel R., Fischer P., Cowburn R.P. (2017). Three-dimensional nanomagnetism. Nat. Commun..

[52] Fischer P., Sanz-Hernández D., Streubel R., Fernández-Pacheco A. (2020). Launching a new dimension with 3D magnetic nanostructures. APL Mater..

[53] Hertel R. (2013). Curvature-induced magnetochirality. SPIN.

[54] Kravchuk V.P., Sheka D.D., Streubel R., Makarov D., Schmidt O.G., Gaididei Y. (2012). Out-of-surface vortices in spherical shells. Phys. Rev. B.

[55] Kravchuk V.P., Rößler U.K., Volkov O.M., Sheka D.D., van den Brink J., Makarov D., Fuchs H., Fangohr H., Gaididei Y. (2016). Topologically stable magnetization states on a spherical shell: Curvature-stabilized skyrmions. Phys. Rev. B.

[56] Jager E.W.H., Inganas O., Lundstrom I. (2000). Microrobots for Micrometer-Size Objects in Aqueous Media: Potential Tools for Single-Cell Manipulation. Science.

[57] Ciuti G., Valdastri P., Menciassi A., Dario P. (2009). Robotic magnetic steering and locomotion of capsule endoscope for diagnostic and surgical endoluminal procedures. Robotica.

[58] Nelson B.J., Kaliakatsos I.K., Abbott J.J. (2010). Microrobots for minimally invasive medicine. Annu. Rev. Biomed. Eng..

[59] Mahoney A.W., Abbott J.J. (2015). Five-degree-of-freedom manipulation of an untethered magnetic device in fluid using a single permanent magnet with application in stomach capsule endoscopy. Int. J. Robot. Res..

[60] Zhan Z., Wei F., Zheng J., Yang W., Luo J., Yao L. (2018). Recent advances of light-driven micro/nanomotors: Toward powerful thrust and precise control. Nanotechnol. Rev..

[61] Jang D., Jeong J., Song H., Chung S.K. (2019). Targeted drug delivery technology using untethered microrobots: A review. J. Micromech. Microeng..

[62] Forbrigger C., Lim A., Onaizah O., Salmanipour S., Looi T., Drake J., Diller E.D. (2019). Cable-Less, Magnetically Driven Forceps for Minimally Invasive Surgery. IEEE Robot. Autom. Lett..

[63] Li J., Wang H., Cui J., Shi Q., Zheng Z., Sun T., Huang Q., Fukuda T. (2019). Magnetic Micromachine Using Nickel Nanoparticles for Propelling and Releasing in Indirect Assembly of Cell-Laden Micromodules. Micromachines.

[64] Ruiz-Gómez S., Foerster M., Aballe L., Proenca M.P., Lucas I., Prieto J., Mascaraque A., de la Figuera J., Quesada A., Pérez L. (2018). Observation of a topologically protected state in a magnetic domain wall stabilized by a ferromagnetic chemical barrier. Sci. Rep..

[65] Reyes D., Biziere N., Warot-Fonrose B., Wade T., Gatel C. (2016). Magnetic Configurations in Co/Cu Multilayered Nanowires: Evidence of Structural and Magnetic Interplay. Nano Lett..

[66] Bran C., Berganza E., Fernandez-Roldan J.A., Palmero E.M., Meier J., Calle E., Jaafar M., Foerster M., Aballe L., Fraile Rodriguez A. (2018). Magnetization Ratchet in Cylindrical Nanowires. ACS Nano.

[67] Wolf D., Biziere N., Sturm S., Reyes D., Wade T., Niermann T., Krehl J., Warot-Fonrose B., Büchner B., Snoeck E. (2019). Holographic vector field electron tomography of three-dimensional nanomagnets. Commun. Phys..

[68] Ivanov Y.P., Chuvilin A., Lopatin S., Kosel J. (2016). Modulated Magnetic Nanowires for Controlling Domain Wall Motion: Toward 3D Magnetic Memories. ACS Nano.

[69] Drisko G.L., Gatel C., Fazzini P.F., Ibarra A., Mourdikoudis S., Bley V., Fajerwerg K., Fau P., Kahn M. (2018). Air-Stable Anisotropic Monocrystalline Nickel Nanowires Characterized Using Electron Holography. Nano Lett..

[70] Andersen I.M., Rodríguez L.A., Bran C., Marcelot C., Joulie S., Hungria T., Vazquez M., Gatel C., Snoeck E. (2020). Exotic Transverse-Vortex Magnetic Configurations in CoNi Nanowires. ACS Nano.

[71] Streubel R., Lee J., Makarov D., Im M.Y., Karnaushenko D., Han L., Schäfer R., Fischer P., Kim S.K., Schmidt O.G. (2014). Magnetic Microstructure of Rolled-Up Single-Layer Ferromagnetic Nanomembranes. Adv. Mater..

[72] Streubel R., Kronast F., Fischer P., Parkinson D., Schmidt O.G., Makarov D. (2015). Retrieving spin textures on curved magnetic thin films with full-field soft X-ray microscopies. Nat. Commun..

[73] Karnaushenko D., Karnaushenko D.D., Makarov D., Baunack S., Schäfer R., Schmidt O.G. (2015). Self-Assembled On-Chip-Integrated Giant Magneto-Impedance Sensorics. Adv. Mater..

[74] Armstrong J.P.K., Stevens M.M. (2020). Using Remote Fields for Complex Tissue Engineering. Trends Biotechnol..

[75] Antman-Passig M., Shefi O. (2016). Remote Magnetic Orientation of 3D Collagen Hydrogels for Directed Neuronal Regeneration. Nano Lett..

[76] Eguchi Y., Ohtori S., Sekino M., Ueno S. (2015). Effectiveness of magnetically aligned collagen for neural regeneration in vitro and in vivo. Bioelectromagnetics.

